# Outcomes of Screening Colonoscopy in Older Adults: A Large Propensity‐Matched Real‐World Cohort Study

**DOI:** 10.1002/jgh3.70479

**Published:** 2026-10-04

**Authors:** Taha Shakarchi, Muneeb Khawar, Mohammad Azeem Malik, Anuhya Jamandlamudi, Ihsan Noushad Karuppan Veettil, Aqsa Komel, Mirza Muhammad Hadeed Khawar, Naima Agha, Muhammad Habib, Ishtiaq Ahmad, Ban Al‐Abayechi, Ahmed Al‐Khazraji

**Affiliations:** ^1^ Lahey Hospital and Medical Center Burlington Massachusetts USA; ^2^ King Edward Medical University Lahore Pakistan; ^3^ Guntur Medical College Guntur Andhra Pradesh India; ^4^ Sree Narayana Institute of Medical Sciences Ernakulam Kerala India; ^5^ Mayo Clinic Rochester Minnesota USA; ^6^ Services Institute of Medical Sciences Lahore Pakistan; ^7^ Foundation University Medical College Rawalpindi Pakistan; ^8^ University of Iowa Hospitals & Clinics Iowa City Iowa USA; ^9^ Rutgers New Jersey Medical School Newark New Jersey USA

**Keywords:** colonoscopy, colorectal cancer screening, mortality, older adults, perforation, propensity score matching, real‐world evidence, TriNetX

## Abstract

**Background:**

The benefits and harms of screening colonoscopy after age 75 remain uncertain. Guidelines endorse individualized decisions given competing risks, but comparative data are limited.

**Methods:**

In the TriNetX US Collaborative Network, adults aged 75–90 years with a colorectal cancer screening encounter were matched 1:1 by colonoscopy within 3 months. Outcomes were assessed at 30 days, 1 year, and 5 years. Sensitivity analyses excluded pre‐index anemia and, separately, fecal abnormalities and alarm symptoms; subsequent colonoscopy was counted through 5 years.

**Results:**

Matched cohorts comprised 58 315 pairs at 30 days, 61 497 at 1 year, and 69 821 at 5 years. Colonoscopy increased polyp detection (HR 4.43 [95% CI 4.15–4.73] at 30 days; 1.88 [1.82–1.95] at 5 years) and colorectal cancer detection (4.56 [3.62–5.74]; 1.78 [1.59–1.98]; all *p* ≤ 0.003). Five‐year all‐cause mortality was unchanged (HR 1.01 [0.97–1.05]; *p* = 0.741). Perforation rose at 5 years (HR 1.29 [1.02–1.63]; *p* = 0.033); gastrointestinal bleeding fell at 1 year (HR 0.90 [0.84–0.97]; *p* = 0.004). After exclusion of pre‐index anemia (70 399 pairs), 5‐year HRs remained 1.72 for polyps and 1.52 for cancer. In an indication‐restricted cohort also excluding fecal abnormalities, weight loss, abdominal pain, and change in bowel habit (54 300 pairs), the 5‐year HRs were 1.59 and 1.32. Subsequent colonoscopy from day 31 to year 5 was not more frequent after index screening (5.8% vs. 6.2%; HR 0.93).

**Conclusion:**

Screening colonoscopy improved polyp and cancer detection but did not reduce 5‐year all‐cause mortality. The late perforation signal was not explained by more subsequent or therapeutic colonoscopy. Decisions after age 75 should be individualized.

## 1 Introduction

Colorectal cancer (CRC) remains the third most diagnosed cancer worldwide [1]. The incidence of this condition rises considerably with age [2]. In the United States, colorectal cancer is the third most common malignancy and the second greatest cause of cancer‐related death [3]. Colonoscopy is considered the gold standard for CRC screening, early identification, and therapeutic polypectomy [4, 5]. However, its clinical value and safety profile in older adults remain highly debated.

The American Cancer Society (ACS) recommends routine screening with a high‐sensitivity stool‐based test or a visual examination for persons 45 years of age and older with an average risk of colorectal cancer (CRC). Regular screening is highly recommended for people 50 years of age and above [6]. Clinical guidelines frequently recommend individualizing screening decisions for patients aged up to 75 years, while the choice to screen for colorectal cancer in persons between the ages of 76 and 85 should be made on an individual basis [7].

These suggestions seek to strike a compromise between the possible long‐term benefits of early cancer identification and the procedure's immediate dangers, which include bowel perforation, gastrointestinal bleeding, and severe electrolyte imbalances following bowel preparation [8]. Despite these guideline frameworks, real‐world practice varies widely. Physicians caring for older patients with multiple comorbidities and widely differing life expectancies face difficult individualized decisions, often with limited high‐quality comparative data to guide them.

Existing studies have documented higher rates of neoplasia in older adults but substantially smaller projected gains in life expectancy compared with younger populations [9, 10]. Large‐scale comparative analyses directly contrasting screened versus unscreened adults aged 75–90 years remain scarce. Such evidence is essential to determine whether the diagnostic advantages of colonoscopy in this age group justify its risks and costs within a clinically realistic time frame. To address this evidence gap, we conducted a large retrospective cohort study using the TriNetX US Collaborative Network to compare clinical outcomes between propensity score‐matched adults aged 75–90 years who underwent screening colonoscopy and those who did not.

## 2 Methods

### 2.1 Data Source and Study Design

We conducted a retrospective cohort study using the TriNetX US Collaborative Network, a federated database of de‐identified electronic health record and claims data from 65 U.S. healthcare organizations. The study population comprised 352 041 patients aged 75–90 years with a colorectal cancer screening encounter between January 1, 2013, and May 1, 2026 (data extracted May 28, 2026). Diagnoses, procedures, and visits were identified using ICD‐10‐CM, CPT, HCPCS, SNOMED CT, and UMLS codes. All data are de‐identified and HIPAA‐compliant; institutional review board approval was not required. The study was reported by STROBE guidelines [11].

### 2.2 Study Population and Exposure Definition

Patients aged 75–90 years with at least one colorectal cancer screening encounter (ICD‐10‐CM: Z12.11 or Z12.12) were eligible. The Colonoscopy group included patients with a colonoscopy procedure (CPT: 45378, 45 380, 45 385 or HCPCS: G0105, G0121) within 3 months of the screening encounter; the No‐colonoscopy group had a screening encounter but no colonoscopy codes in that window. Patients with gastrointestinal hemorrhage (K92.0–K92.2) within 1 month before the index date, or prior colon polyps (K63.5, Z86.010), colorectal cancer (C18–C20), or inflammatory bowel disease (K50–K51) recorded at least 1 month before, were excluded from both groups (detailed in Tables S1 and S2). The index date was the first eligible screening encounter; patients were followed from 1 day after until the outcome, death, or last recorded fact. Eligibility required a dedicated colorectal cancer screening encounter code (Z12.11 or Z12.12). Two additional sensitivity cohorts were defined to reduce indication contamination. An anemia‐excluded cohort applied the same rules and additionally excluded anemia (ICD‐10‐CM D64.9) recorded before the screening encounter. An indication‐restricted (“strict screening”) cohort further excluded fecal abnormalities (R19.5), unexplained weight loss (R63.4), unspecified abdominal pain (R10.9), and change in bowel habit (R19.4) in the 6 months before the screening encounter.

### 2.3 Outcomes

We assessed all‐cause mortality and seven clinical outcomes at 30 days, 1 year, and 5 years: non‐traumatic intestinal perforation, gastrointestinal bleeding, electrolyte abnormalities, inpatient hospitalization, emergency department visits, polyp detection, and incident colorectal cancer. Outcome code lists and analysis settings are in Table S3. Outcomes were identified using ICD‐10‐CM, CPT/HCPCS, SNOMED CT, and HL7V3.0 codes. Kaplan–Meier and risk analyses included patients with the outcome recorded prior to the observation window; frequency analyses for count outcomes (hospitalization, emergency department visits, gastrointestinal bleeding) excluded patients with zero events. All‐cause mortality was prespecified as an efficacy endpoint alongside diagnostic yield and safety. Colorectal cancer stage and cancer‐specific mortality were not available in the platform. To examine whether late perforation could be explained by downstream procedures, we additionally measured any subsequent colonoscopy and therapeutic colonoscopy (CPT 45380 or 45 385) from day 1 to day 1825 and, as the primary subsequent‐procedure window, from day 31 to day 1825 after the index date.

### 2.4 Covariates and Propensity Score Matching

We performed 1:1 propensity score matching separately for each follow‐up window (30‐day, 1‐year, and 5‐year) to ensure inclusion of patients with sufficient observation time. Covariates included demographics (age, sex, race/ethnicity), comorbidities (essential hypertension, type 2 diabetes mellitus, chronic kidney disease, heart failure, coronary artery disease, atrial fibrillation, COPD, stroke, dementia, obesity, cirrhosis, anemia, and tobacco use), and medications (aspirin, clopidogrel, warfarin, apixaban, rivaroxaban, dabigatran, and long‐term NSAIDs). Propensity scores were estimated by logistic regression [12, 13]; matching used a nearest‐neighbor algorithm with a caliper of 0.1 pooled SDs. Post‐matching balance was assessed via standardized mean differences and *p*‐values (Tables S4a–c). After matching, the analytic sample sizes were 58 315 per group for 30‐day analyses, 61 497 per group for 1‐year analyses, and 69 821 per group for 5‐year analyses.

### 2.5 Statistical Analysis

All analyses used validated TriNetX platform modules. Cox proportional hazards models estimated hazard ratios (HRs) with 95% confidence intervals; Kaplan–Meier curves and log‐rank tests compared cumulative incidence between matched groups. For repeated‐event outcomes (hospitalizations, emergency department visits, gastrointestinal bleeding), frequency analyses compared group means by *t*‐test. Statistical significance was defined as two‐sided *p* < 0.05; no correction for multiple comparisons was applied.

### 2.6 Sensitivity and Subgroup Analyses

We performed pre‐specified sensitivity and subgroup analyses to evaluate the robustness of the primary findings. A strict‐definition sensitivity analysis limited the population to patients aged 75–85 years and defined colonoscopy exposure using CPT codes only (excluding HCPCS screening codes). Subgroup analyses were stratified by sex and by age bands (75–79, 80–84, and 85–90 years). Two post hoc analyses were added in response to peer review. First, diagnostic yield (polyp detection and colorectal cancer) was re‐estimated after exclusion of pre‐index anemia and again in the indication‐restricted cohort that also excluded fecal abnormalities and coded alarm symptoms. Second, the frequency of any subsequent colonoscopy and of therapeutic colonoscopy was compared between matched groups through 5 years. All sensitivity and subgroup analyses used identical 1:1 propensity score matching.

## 3 Results

### 3.1 Study Population

Of 352 041 eligible patients aged 75–90 years identified from the TriNetX US Collaborative Network, 72 225 underwent colonoscopy screening and 279 816 did not. After excluding patients with prior colorectal cancer, colon polyps, inflammatory bowel disease, or gastrointestinal hemorrhage, 1:1 propensity score matching yielded 58 315 pairs for the 30‐day analysis, 61 497 pairs for the 1‐year analysis, and 69 821 pairs for the 5‐year analysis (Figure 1).

**FIGURE 1:**
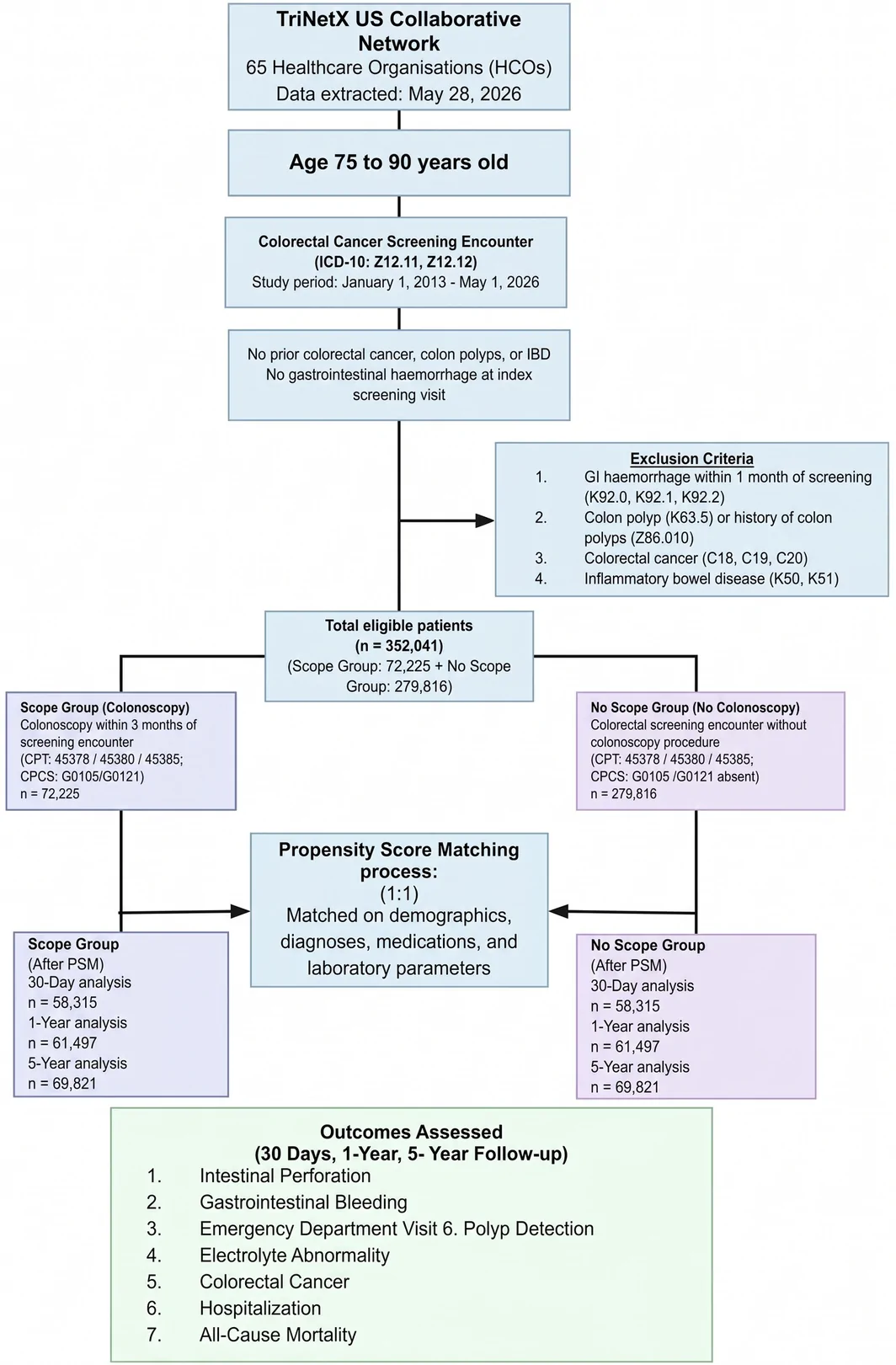
Flowchart of patient selection.

### 3.2 Baseline Characteristics

After matching, both cohorts were well balanced. Mean age was 77.4 ± 2.5 versus 77.4 ± 2.6 years; females comprised 53.6% versus 54.2%; and White patients 81.4% versus 81.6%, respectively. Comorbidity prevalence was comparable: essential hypertension (66.6% vs. 65.7%), type 2 diabetes mellitus (25.5% vs. 24.8%), chronic kidney disease (16.0% vs. 15.2%), coronary artery disease (23.6% vs. 22.4%), atrial fibrillation (12.0% vs. 10.9%), and COPD (10.1% vs. 9.2%). Medication was similarly balanced (Table 1).

**TABLE 1:** Baseline Characteristics of Patients Aged 75–90 Years Undergoing Colonoscopy Screening (Colonoscopy Group) versus No Colonoscopy (No Colonoscopy Group) Before and After Propensity Score Matching.

Variable	Before propensity score matching			After propensity score matching		
	Cohort 1 colonoscopy group (*n* = 58 322)	Cohort 2 no colonoscopy group (*n* = 248 087)	*p*	Cohort 1 colonoscopy group (*n* = 58 315)	Cohort 2 no colonoscopy group (*n* = 58 315)	*p*
*Demographics*						
Age at index event, mean ± SD (years)	77.4 ± 2.5	77.2 ± 2.6	< 0.001	77.4 ± 2.5	77.4 ± 2.6	0.843
Age at index event, median (IQR)	77 (4)	76 (4)		77 (4)	77 (4)	
Female sex	31 277 (53.6%)	148 748 (60.0%)	< 0.001	31 274 (53.6%)	31 590 (54.2%)	0.063
Male sex	27 033 (46.4%)	99 219 (40.0%)	< 0.001	27 029 (46.3%)	26 695 (45.8%)	0.050
White	47 490 (81.4%)	191 794 (77.3%)	< 0.001	47 483 (81.4%)	47 565 (81.6%)	0.536
Black or African American	5277 (9.0%)	27 984 (11.3%)	< 0.001	5277 (9.0%)	5275 (9.0%)	0.984
Hispanic or Latino	2926 (5.0%)	8593 (3.5%)	< 0.001	2921 (5.0%)	2954 (5.1%)	0.659
Asian	2410 (4.1%)	10 347 (4.2%)	0.675	2410 (4.1%)	2399 (4.1%)	0.871
Other Race	893 (1.5%)	6792 (2.7%)	< 0.001	893 (1.5%)	875 (1.5%)	0.666
Unknown Race	1923 (3.3%)	9538 (3.8%)	< 0.001	1923 (3.3%)	1888 (3.2%)	0.564
*Comorbid diagnoses*						
Essential hypertension (I10)	38 868 (66.6%)	171 090 (69.0%)	< 0.001	38 864 (66.6%)	38 315 (65.7%)	0.001
Type 2 diabetes mellitus (E11)	14 845 (25.5%)	66 807 (26.9%)	< 0.001	14 844 (25.5%)	14 480 (24.8%)	0.014
Chronic kidney disease (N18)	9322 (16.0%)	41 757 (16.8%)	< 0.001	9322 (16.0%)	8842 (15.2%)	< 0.001
Heart failure (I50)	5711 (9.8%)	23 742 (9.6%)	0.101	5709 (9.8%)	5251 (9.0%)	< 0.001
Chronic obstructive pulmonary disease (J44)	5881 (10.1%)	25 174 (10.1%)	0.647	5880 (10.1%)	5351 (9.2%)	< 0.001
Coronary artery disease (I25.1)	13 781 (23.6%)	51 615 (20.8%)	< 0.001	13 777 (23.6%)	13 042 (22.4%)	< 0.001
Atrial fibrillation (I48.91)	6973 (12.0%)	26 245 (10.6%)	< 0.001	6971 (12.0%)	6334 (10.9%)	< 0.001
Cerebral infarction/stroke (I63)	2891 (5.0%)	13 504 (5.4%)	< 0.001	2891 (5.0%)	2665 (4.6%)	0.002
Unspecified dementia (F03)	1029 (1.8%)	5425 (2.2%)	< 0.001	1029 (1.8%)	941 (1.6%)	0.046
Overweight and obesity (E66)	12 931 (22.2%)	56 084 (22.6%)	0.024	12 927 (22.2%)	12 337 (21.2%)	< 0.001
Unspecified cirrhosis of liver (K74.60)	876 (1.5%)	2163 (0.9%)	< 0.001	870 (1.5%)	830 (1.4%)	0.328
Anemia, unspecified (D64.9)	10 758 (18.4%)	43 736 (17.6%)	< 0.001	10 755 (18.4%)	10 090 (17.3%)	< 0.001
Tobacco use (Z72.0)	1767 (3.0%)	8722 (3.5%)	< 0.001	1767 (3.0%)	1490 (2.6%)	< 0.001
Long‐term NSAID Use (Z79.1)	2313 (4.0%)	6433 (2.6%)	< 0.001	2307 (4.0%)	2196 (3.8%)	0.092
*Medications*						
Aspirin	18 075 (31.0%)	73 482 (29.6%)	< 0.001	18 070 (31.0%)	17 456 (29.9%)	< 0.001
Clopidogrel	4359 (7.5%)	18 427 (7.4%)	0.701	4358 (7.5%)	3929 (6.7%)	< 0.001
Warfarin	2893 (5.0%)	12 154 (4.9%)	0.538	2893 (5.0%)	2664 (4.6%)	0.002
Apixaban	4360 (7.5%)	16 552 (6.7%)	< 0.001	4359 (7.5%)	3983 (6.8%)	< 0.001
Rivaroxaban	1856 (3.2%)	7216 (2.9%)	< 0.001	1855 (3.2%)	1749 (3.0%)	0.073
Dabigatran	26 (0.0%)	59 (0.0%)	0.007	26 (0.0%)	15 (0.0%)	0.086

*Note:* Data represents *n* (%) unless otherwise indicated. Cohort 1 = colonoscopy group (colonoscopy with CPT 45378/45380/45385 or HCPCS G0105/G0121); Cohort 2 = no colonoscopy group (colorectal cancer screening without colonoscopy procedure). Study period: January 1, 2013–May 1, 2026. Network: TriNetX US Collaborative Network (65 HC).

Abbreviations: CAD, coronary artery disease; COPD, chronic obstructive pulmonary disease; IQR, interquartile range; NSAID, non‐steroidal anti‐inflammatory drug; PSM, propensity score matching.

### 3.3 Clinical Outcomes

Colonoscopy was associated with substantially higher diagnostic yield at every follow‐up interval. Polyp detection HRs declined from 4.43 (95% CI 4.15–4.73) at 30 days to 2.16 (95% CI 2.08–2.25) at 1 year and 1.88 (95% CI 1.82–1.95) at 5 years (all *p* < 0.001); 5‐year event rates were 14.07% versus 7.92%. Colorectal cancer detection followed a parallel pattern: HR 4.56 (95% CI 3.62–5.74) at 30 days, 2.22 (95% CI 1.92–2.55) at 1 year, and 1.78 (95% CI 1.59–1.98) at 5 years (all *p* ≤ 0.003). Kaplan–Meier curves showed early and sustained separation in detection. All‐cause mortality was similar between groups at all horizons (30‐day HR 0.98, *p* = 0.929; 1‐year HR 0.94, *p* = 0.162; 5‐year HR 1.01, 95% CI 0.97–1.05, *p* = 0.741), with superimposable survival curves (Figure S7).

Safety outcomes showed a mixed, time‐dependent profile. Intestinal perforation was non‐significantly higher at 30 days (HR 1.58, *p* = 0.126) and 1 year (HR 1.29, *p* = 0.150), reaching significance at 5 years (0.23% vs. 0.18%; HR 1.29, 95% CI 1.02–1.63; *p* = 0.033), with late Kaplan–Meier curve divergence (Figure 2). Gastrointestinal bleeding was modestly lower at 1 year (HR 0.90, *p* = 0.004) but similar otherwise. Electrolyte abnormalities did not differ significantly (30‐day HR 0.89, *p* = 0.051; 5‐year HR 1.03, *p* = 0.067). Hospitalization was consistently lower after colonoscopy (30‐day HR 0.78, 95% CI 0.73–0.84; 1‐year HR 0.90, 95% CI 0.87–0.93; 5‐year HR 0.96, 95% CI 0.94–0.97; all *p* < 0.001), while emergency department visits were consistently higher (HRs ≈ 1.19; all *p* < 0.001). The 5‐year forest plot is shown in Figure 3 (For 30‐day and 1 year follow up, forest plots are in Figures S8 and S9). These contrasting utilization patterns are visualized in Kaplan–Meier plots (Figure 2 and Figure S7). Complete event counts, HRs, CIs, and *p*‐values are in Table 2.

**FIGURE 2:**
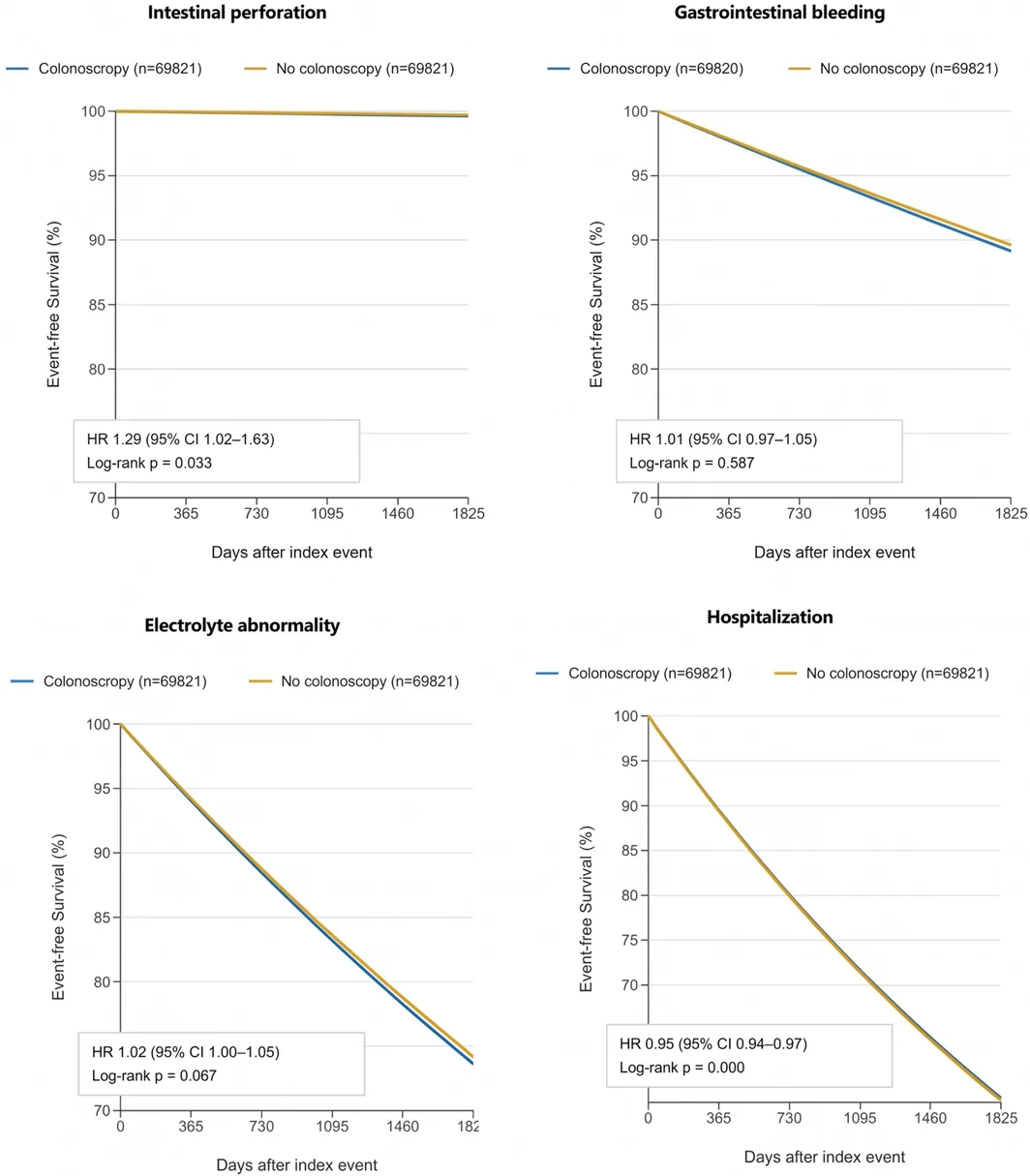
Kaplan–Meier survival curves for intestinal perforation, gastrointestinal bleed, electrolyte abnormality, and hospitalization compared with the no‐colonoscopy group.

**FIGURE 3:**
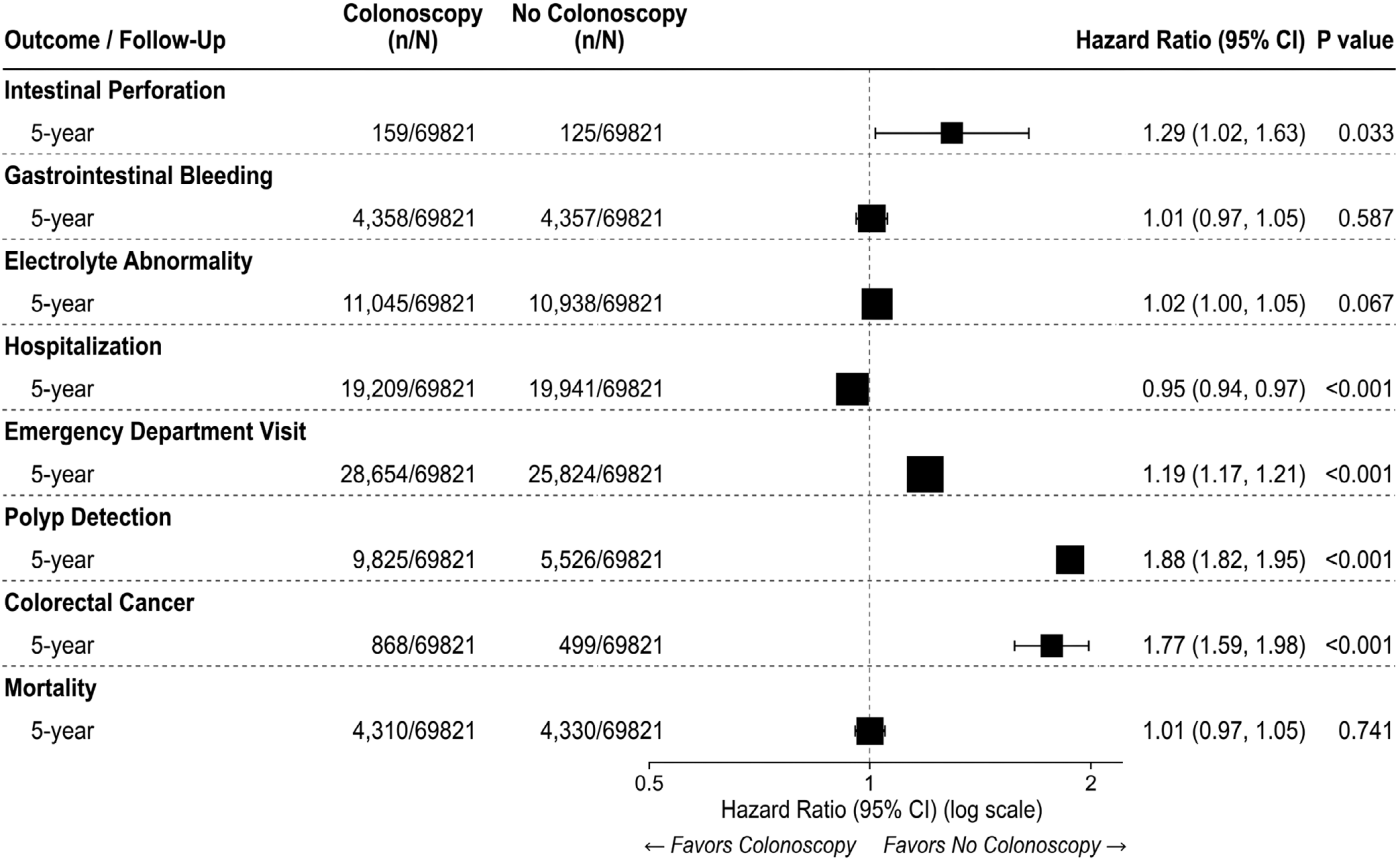
Forest Plot of pooled effect for 5‐year clinical outcome of intestinal perforation, gastrointestinal bleed, electrolyte abnormality and hospitalization, emergency department visit, polyp detection, colorectal cancer and mortality.

**TABLE 2:** Clinical outcomes in patients aged 75–90 years undergoing colonoscopy screening (colonoscopy group) versus no colonoscopy (no colonoscopy group) at 30‐day, 1‐year, and 5‐year follow‐up after propensity score matching.

Outcome	Colonoscopy group events, *n* (%)	No colonoscopy group events, *n* (%)	Hazard ratio (95% CI)	*p*
	(*n* = 58 315)	(*n* = 58 315)		
*30‐day follow‐up*				
Intestinal perforation	28 (0.05%)	18 (0.03%)	1.58 (0.88–2.86)	0.126
Gastrointestinal bleeding	372 (0.64%)	421 (0.72%)	0.90 (0.78–1.04)	0.139
Electrolyte abnormality	546 (0.94%)	624 (1.07%)	0.89 (0.80–1.00)	0.051
Hospitalization	1562 (2.68%)	2025 (3.47%)	0.78 (0.73–0.84)	< 0.001
Emergency department visit	1701 (2.92%)	1459 (2.50%)	1.19 (1.11–1.28)	< 0.001
Polyp detection	4808 (8.24%)	1142 (1.96%)	4.43 (4.15–4.73)	< 0.001
Colorectal cancer	397 (0.68%)	89 (0.15%)	4.56 (3.62–5.74)	0.003
Mortality	49 (0.08%)	51 (0.09%)	0.98 (0.66–1.45)	0.929
	(*n* = 61 497)	(*n* = 61 497)		
*1‐year follow‐up*				
Intestinal perforation	71 (0.12%)	56 (0.09%)	1.29 (0.91–1.83)	0.150
Gastrointestinal bleeding	1528 (2.48%)	1722 (2.80%)	0.90 (0.84–0.97)	0.004
Electrolyte abnormality	3977 (6.47%)	4002 (6.51%)	1.01 (0.97–1.06)	0.551
Hospitalization	8489 (13.81%)	9520 (15.48%)	0.90 (0.87–0.93)	< 0.001
Emergency department visit	12 484 (20.30%)	10 904 (17.73%)	1.19 (1.16–1.22)	< 0.001
Polyp detection	7058 (11.48%)	3485 (5.67%)	2.16 (2.08–2.25)	< 0.001
Colorectal cancer	610 (0.99%)	282 (0.46%)	2.22 (1.92–2.55)	< 0.001
Mortality	844 (1.37%)	920 (1.50%)	0.94 (0.85–1.03)	0.162
	(*n* = 69 821)	(*n* = 69 821)		
*5‐year follow‐up*				
Intestinal perforation	159 (0.23%)	125 (0.18%)	1.29 (1.02–1.63)	0.033
Gastrointestinal bleeding	4358 (6.24%)	4357 (6.24%)	1.01 (0.97–1.06)	0.587
Electrolyte abnormality	11 045 (15.82%)	10 938 (15.67%)	1.03 (1.00–1.05)	0.067
Hospitalization	19 209 (27.51%)	19 941 (28.56%)	0.96 (0.94–0.97)	< 0.001
Emergency department visit	28 654 (41.04%)	25 824 (36.99%)	1.19 (1.17–1.21)	< 0.001
Polyp detection	9825 (14.07%)	5526 (7.92%)	1.88 (1.82–1.95)	< 0.001
Colorectal cancer	868 (1.24%)	499 (0.71%)	1.78 (1.59–1.98)	< 0.001
Mortality	4310 (6.17%)	4330 (6.20%)	1.01 (0.97–1.05)	0.741

*Note:* PSM cohort sizes differ by follow‐up window as TriNetX restricts inclusion to patients with sufficient data coverage for each time horizon (30‐day: *n* = 58 315 per cohort; 1‐year: *n* = 61 497 per cohort; 5‐year: *n* = 69 821 per cohort). Outcomes derived from TriNetX Kaplan–Meier survival analysis. *p* from log‐rank test. All analyses performed on propensity score–matched cohorts.

Abbreviations: CI, confidence interval; HR, hazard ratio.

### 3.4 Sensitivity and Subgroup Analyses

Sensitivity and subgroup analyses were performed to assess the robustness of the primary findings (Table S5). A strict‐definition sensitivity analysis (ages 75–85, CPT codes only; 37 434 matched pairs) confirmed higher polyp detection, colorectal cancer detection, and emergency department visits. However, electrolyte abnormalities were significantly more frequent with colonoscopy (HR 1.08, 95% CI 1.04–1.12; *p* < 0.001), all‐cause mortality was modestly higher (HR 1.08, 95% CI 1.01–1.14; *p* = 0.019), and the hospitalization benefit was no longer significant. Intestinal perforation and gastrointestinal bleeding did not differ.

Sex‐stratified analyses showed consistent patterns for diagnostic yield and emergency department utilization. Among females (37 558 per group), colonoscopy was associated with lower hospitalization (HR 0.94, *p* < 0.001), higher emergency department visits, and higher polyp and colorectal cancer detection, with no mortality difference. Among males (33 270 per group), only emergency department visits and detection outcomes were significantly higher; hospitalization, perforation, bleeding, electrolyte, and mortality differences were non‐significant.

Age‐stratified analyses for the 75–79 and 80–84‐year strata largely mirrored the primary findings for detection and emergency department visits. In the 85–90‐year stratum, the analysis did not return valid effect estimates: the platform reported a hazard ratio of exactly 1.000 for every outcome, including outcomes with clearly different underlying event frequencies. This degenerate pattern is incompatible with a true biological effect and instead reflects a limitation of the analytic platform in the oldest and smallest stratum, where low event counts approach the platform's low‐count obfuscation threshold and can force identical reported event totals in both arms. We therefore treat this stratum as uninterpretable rather than as evidence of a null effect; it is reported in Table S5 for completeness only and is excluded from all clinical inference. The primary findings rest on the 75–79 and 80–84 strata, which were unaffected. Kaplan–Meier curves for all sensitivity and subgroup analyses are in Figures S1–S6.

In a separately matched extract that excluded anemia recorded before the index screening encounter (73 healthcare organizations; 70 399 pairs), diagnostic yield remained higher in the colonoscopy arm at every horizon: 30‐day polyp detection 6.83% versus 2.05% (HR 3.51, 95% CI 3.31–3.72; *p* < 0.001) and colorectal cancer 0.58% versus 0.16% (HR 3.81, 95% CI 3.09–4.70; *p* < 0.001); 1‐year polyp detection HR 1.75 (95% CI 1.69–1.82) and colorectal cancer HR 2.02 (95% CI 1.76–2.32); and 5‐year polyp detection 13.50% versus 8.34% (HR 1.72, 95% CI 1.66–1.78; *p* < 0.001) and colorectal cancer 1.13% versus 0.77% (HR 1.52, 95% CI 1.36–1.69; *p* < 0.001) (Table S6, Panel A). In the more restrictive indication‐restricted extract, which additionally excluded fecal abnormalities, unexplained weight loss, unspecified abdominal pain, and change in bowel habit in the 6 months before screening (70 healthcare organizations; 54 300 pairs), yield was attenuated but remained significantly higher: 30‐day polyp detection 5.54% versus 1.93% (HR 3.02, 95% CI 2.81–3.24; *p* < 0.001) and colorectal cancer 0.47% versus 0.18% (HR 2.74, 95% CI 2.17–3.47; *p* < 0.001); 1‐year polyp detection HR 1.61 (95% CI 1.54–1.68) and colorectal cancer HR 1.77 (95% CI 1.50–2.08); and 5‐year polyp detection 12.00% versus 7.97% (HR 1.59, 95% CI 1.53–1.66; *p* < 0.001) and colorectal cancer 0.99% versus 0.77% (HR 1.32, 95% CI 1.16–1.50; *p* < 0.001) (Table S6, Panel B).

Subsequent colonoscopy was not more common after index screening. In a separately matched extract of the original exposure definition (73 healthcare organizations; 77 504 pairs), any colonoscopy from day 1 to day 1825 occurred in 5137 patients (6.63%) in the colonoscopy arm and 6214 (8.02%) in the no‐colonoscopy arm (HR 0.83, 95% CI 0.80–0.86; log‐rank *p* < 0.001). Restricting the window to day 31–1825, to avoid counting the index examination, any subsequent colonoscopy occurred in 4472 patients (5.77%) versus 4824 (6.22%) (HR 0.93, 95% CI 0.90–0.97; log‐rank *p* = 0.001). Therapeutic colonoscopy (CPT 45380 or 45385) from day 31 to 1825 occurred in 3303 patients (4.26%) versus 3389 (4.37%) (HR 0.99, 95% CI 0.94–1.03; log‐rank *p* = 0.567) (Table S7).

## 4 Discussion

In this large, real‐world, propensity‐matched cohort comprising between 58 315 and 69 821 matched pairs of adults aged 75–90 years, screening colonoscopy was associated with substantially higher detection of colorectal polyps and cancer at every time horizon yet conferred no measurable reduction in all‐cause mortality over 5 years. The diagnostic advantage was greatest immediately after the procedure and attenuated progressively over time, with the polyp‐detection HR falling from 4.43 at 30 days to 1.88 at 5 years, and the colorectal cancer‐detection HR from 4.56 to 1.78, a pattern consistent with early clearance of a prevalent pool of asymptomatic lesions followed by a narrower difference in incident disease. Yield remained higher after exclusion of pre‐index anemia and after further exclusion of fecal abnormalities and coded alarm symptoms. Against this diagnostic benefit, colonoscopy carried a modest, time‐dependent excess of intestinal perforation that reached statistical significance only at 5 years (HR 1.29), alongside a consistent increase in emergency‐department utilization but, paradoxically, fewer hospitalisations. Subsequent colonoscopy after day 31 was not more frequent in the screened arm. Sensitivity and subgroup analyses broadly reproduced these patterns but uncovered a small yet significant excess mortality in the strict‐definition cohort, underscoring the fragility of the benefit–risk balance in this age group. Taken together, these findings suggest that the value of colonoscopy in the very elderly lies chiefly in diagnostic yield rather than in a demonstrable survival advantage within a clinically realistic follow‐up window.

The high diagnostic yield we observed is consistent with the established epidemiology of colorectal neoplasia, whose prevalence rises steeply with advancing age [2, 3]. In their seminal cross‐sectional study of 1244 asymptomatic adults, Lin and colleagues reported that the prevalence of colonic neoplasia increased from 13.8% in those aged 50–54 years to 26.5% at 75–79 years and 28.6% in those aged 80 years and older [9]. Our five‐year polyp‐detection rate of 14.07% in the colonoscopy arm versus 7.92% among unscreened patients, and colorectal cancer detection of 1.24% versus 0.71%, are concordant with the expectation that a single colonoscopy in an older adult's population will uncover a meaningful burden of previously occult disease. The same direction of effect persisted after we excluded coded anemia and, in a further restriction, fecal abnormalities and alarm symptoms: five‐year polyp‐detection HRs were 1.72 and 1.59, and five‐year colorectal‐cancer HRs were 1.52 and 1.32, respectively. Attenuation relative to the primary analysis is consistent with some diagnostic examinations having been coded as screening in the main cohort, but the residual excess detection indicates that yield is not explained solely by that misclassification. The marked attenuation of the detection hazard ratio over time, from more than fourfold at 30 days to less than twofold at 5 years, most plausibly reflects the difference between prevalent and incident detection: colonoscopy rapidly identifies lesions already present at the index date (a one‐time “harvest”), after which the gap between groups narrows as unscreened patients accrue diagnoses through symptomatic presentation and incidental testing. This phenomenon, partly attributable to lead‐time, cautions against interpreting the early, dramatic hazard ratios as evidence of a proportionate long‐term clinical benefit.

Perhaps the most clinically consequential finding is the absence of any all‐cause mortality benefit at 30 days, 1 year, or 5 years (five‐year HR 1.01, 95% CI 0.97–1.05; *p* = 0.741). We retained all‐cause mortality as a primary endpoint deliberately, while emphasizing that a null result was anticipated rather than surprising: it is the patient‐centred outcome that ultimately matters to older adults weighing whether to be screened, yet it is intrinsically insensitive to a colorectal‐cancer intervention in this age group and should not be read as a direct test of the adenoma–carcinoma efficacy of colonoscopy. This aligns closely with the best available randomized and quasi‐experimental evidence. In the landmark NordICC trial of 84 585 adults aged 55–64 years, an invitation to colonoscopy reduced the 10‐year risk of colorectal cancer from 1.20% to 0.98% (risk ratio 0.82) but produced no significant reduction in colorectal cancer‐related death (0.28% vs. 0.31%; RR 0.90, 95% CI 0.64–1.16) and no effect on all‐cause mortality [5]; longer‐term follow‐up of the same cohort has continued to show a reduction in cancer incidence of approximately 30% with no all‐cause mortality signal [14]. Even in per‐protocol analyses adjusted for actual colonoscopy uptake, the projected colorectal cancer mortality reduction translated into only a very small absolute benefit [5]. The age‐specific data are still more sobering: in the emulated target trial by García‐Albéniz and colleagues of 1.36 million Medicare beneficiaries, screening colonoscopy reduced the 8‐year risk of colorectal cancer by an absolute 0.42% among those aged 70–74 years but by only a non‐significant 0.14% among those aged 75–79 years (2.84% vs. 2.97%; risk difference −0.14%, 95% CI −0.41 to 0.16) [6]. Lin et al. likewise estimated that the mean gain in life expectancy from screening colonoscopy in patients aged 80 years and older was just 0.13 years, compared with 0.85 years in 50–54‐year‐olds, only about 15% of the younger benefit [9]. Several mechanisms explain why our null mortality result is expected rather than surprising. First, all‐cause mortality is an insensitive endpoint in this setting, because colorectal cancer accounts for only a small fraction of deaths in older adults, whose competing risks from cardiovascular, pulmonary, renal and neurodegenerative disease dominate. Second, the survival benefit of screening accrues over a 10‐ to 15‐year horizon that exceeds both our five‐year window and the life expectancy of many patients in this cohort; El Halabi et al. demonstrated that the majority of screening colonoscopies performed in adults older than 75 years occur in individuals with an estimated life expectancy of less than 10 years, in whom colorectal cancer is rarely detected and the time to benefit is unlikely to be realized [4]. Third, lead‐time and the detection of indolent, slow progressive lesions further dilute any survival signal.

The safety profile we observed reinforces the principle that procedural risk, unlike benefit, does not diminish with age. Although perforation was numerically more frequent in the colonoscopy arm at every time point, the difference reached significance only at 5 years, with Kaplan–Meier curves diverging late. A delayed rise in perforation cannot be attributed to the index instrument passage itself. We therefore measured subsequent procedures directly. From day 31 to year 5, any colonoscopy was slightly less common after index screening than after screening without colonoscopy (5.77% vs. 6.22%; HR 0.93, 95% CI 0.90–0.97), and therapeutic colonoscopy did not differ (4.26% vs. 4.37%; HR 0.99, 95% CI 0.94–1.03). A simple volume‐based “cascade” of extra surveillance or therapeutic examinations therefore does not account for the 5‐year perforation association. Remaining explanations include greater procedural complexity among those who do return for later examination, delayed capture of complications in claims data, residual confounding, or chance around a rare event (159 vs. 125 perforations at 5 years). We treat the late signal as an association that is not explained by a higher count of subsequent procedures, not as proof that the index examination caused delayed mechanical injury. Our estimate is consistent in direction and magnitude with the wider literature on age and colonoscopy harm. In the meta‐analysis by Day et al., the pooled perforation rate rose from 1.0 per 1000 colonoscopies in adults aged 65 years and older to 1.5 per 1000 among octogenarians, with the risk of perforation 1.6‐fold higher in those aged 80 years or more than in younger patients (incidence rate ratio 1.6, 95% CI 1.2–2.1) [15]. The more recent meta‐analysis by Lu and colleagues similarly found that adults older than 80 years had a 2.55‐fold higher risk of perforation (RR 2.55, 95% CI 1.15–5.66) and a 23% higher risk of bleeding (RR 1.23, 95% CI 1.02–1.48) than those aged 65–80 years [16]. García‐Albéniz et al. quantified this gradient prospectively, reporting an excess 30‐day adverse‐event rate of 10.3 per 1000 in the 75–79‐year group, nearly double the 5.6 per 1000 observed at 70–74 years [17]. The modestly lower gastrointestinal bleeding rate we found at 1 year is most plausibly explained by the identification and endoscopic treatment of bleeding‐prone lesions, such as angiodysplasia and large polyps, at the index colonoscopy, together with a degree of selection of healthier patients into the screening arm.

The divergent healthcare‐utilization findings, consistently lower hospitalization but higher emergency‐department use after colonoscopy, merit careful interpretation. A lower hospitalization rate following an invasive procedure is counter‐intuitive and is unlikely to represent a direct biological effect of colonoscopy. It most probably reflects residual confounding by indication, or “healthy‐user” bias: despite rigorous matching on documented comorbidities and medications, patients selected for colonoscopy in routine practice are typically fitter, more functionally independent and have longer anticipated survival than those who forgo it, characteristics such as frailty, functional status, cognitive impairment and true life expectancy that are poorly captured in claims‐based data and therefore incompletely balanced by propensity scoring. This is not a peripheral caveat but the central threat to the internal validity of every comparative estimate we report: if the screened cohort is systematically fitter, then the lower hospitalization, the null mortality, and part of the complication differences could be generated by selection alone, independent of any true effect of colonoscopy. The higher emergency‐department utilization, by contrast, is biologically coherent: it captures post‐procedural complaints (abdominal pain, bleeding, dehydration and bowel‐preparation‐related symptoms) and the downstream cascade of evaluations triggered by positive findings. This pattern echoes the work of Grossberg et al., who showed that adults older than 75 years are at significantly increased risk of emergency‐department visits and hospitalization in the period following colonoscopy [8]. Electrolyte disturbance, although not significant in our primary analysis, became significantly more frequent in the colonoscopy arm in the stricter sensitivity analysis (HR 1.08; *p* < 0.001), consistent with the recognized risk of bowel‐preparation‐induced fluid and electrolyte shifts in older patients with reduced renal reserve [18, 19].

Our subgroup and sensitivity analyses add important nuance. In the strict‐definition cohort (ages 75–85, exposure defined by CPT codes only), all‐cause mortality was modestly but significantly higher in the colonoscopy group, a signal that may reflect procedural harm concentrated among less robust patients, residual confounding, or the play of chance across multiple unadjusted comparisons. The uniform hazard ratio of exactly 1.000 across every endpoint in the 85–90‐year stratum is not a credible biological result; identical estimates for perforation, bleeding, detection, utilization, and death alike reflect a platform limitation rather than a true effect. In the oldest and smallest stratum, low event counts fall at or below the platform's low‐count obfuscation threshold, which can return identical reported event totals, and hence unity hazard ratios, in both arms. We have accordingly relegated this stratum to the Supplement and excluded it from interpretation, and it should not be read as demonstrating that colonoscopy is without effect in the very oldest patients.

Collectively, these observations support the position taken by current guidelines and commentators that colonoscopy after age 75 should not be applied routinely on the basis of age alone. The US Preventive Services Task Force recommends individualized, selective screening for adults aged 76–85 years [7], and Dorsey and Roper, among others, have argued that the decision in older adults should be driven by life expectancy, comorbidity burden, prior screening history, and patient preference rather than chronological age [20]. Reassuringly, Dalmat et al. found that the risk of colorectal cancer remains low for many years after a negative colonoscopy even among adults aged 76–85 years, suggesting that previously well‐screened older adult patients derive little incremental benefit from continued examination [21]. Our data, showing tangible diagnostic yield but no survival gain and a rising late harm, reinforce a strategy of shared decision‐making in which the prevalent burden of neoplasia is weighed against competing mortality, procedural risk, and the patient's own goals of care. Consistent with this, the American College of Gastroenterology's 2021 guideline restricts routine screening to ages 50–75 and directs that decisions beyond 75 be individualized on the basis of estimated life expectancy, comorbidity burden, and prior screening history rather than chronological age [22], a framework that directly contextualizes the healthy‐user selection evident in our cohort.

## 5 Limitations

Several limitations temper our conclusions. First, as a retrospective analysis of de‐identified electronic health‐record and claims data, the study remains susceptible to residual and unmeasured confounding, particularly by frailty, functional status and life expectancy, and to the health‐user bias discussed above, which propensity matching cannot fully eliminate. We regard this residual confounding by fitness, rather than any single procedural risk, as the principal threat to the internal validity of our comparative estimates. Second, exposure and outcome ascertainment relied on diagnostic and procedural codes, which are subject to misclassification, including potential blurring of screening and diagnostic colonoscopy. Claims data cannot fully exclude examinations prompted by occult bleeding or altered bowel habits that are coded as screening. Exclusion of pre‐index anemia, fecal abnormalities, and coded alarm symptoms reduced—but did not abolish—the excess of polyp and cancer diagnoses, so residual indication contamination remains possible. Third, the platform did not capture colorectal cancer stage or cancer‐specific mortality, limiting our ability to distinguish the detection of clinically important cancers from indolent disease, and our five‐year horizon is shorter than the interval over which screening benefit typically emerges. In particular, the higher colorectal‐cancer detection we observed (five‐year HR 1.78 in the primary analysis; 1.32 in the indication‐restricted analysis) cannot separate the prevention of fatal interval cancers from the overdiagnosis of indolent lesions that would never have become symptomatic within these patients' remaining lifespan, so part of the excess detection may represent overdiagnosis rather than clinical benefit. Fourth, the anemia‐excluded, indication‐restricted, and subsequent‐procedure analyses were run on later extracts of the US Collaborative Network (70 or 73 healthcare organizations) than the primary analysis (65 organizations) and are therefore not nested within Table 2; they should be read as complementary sensitivity estimates, not as re‐analyses of the identical matched pairs. Fifth, tests of the proportional‐hazards assumption were violated for subsequent‐colonoscopy outcomes, so those hazard ratios represent average effects over follow‐up rather than a constant instantaneous hazard. Sixth, no correction was made for multiple comparisons, so borderline associations, such as the electrolyte abnormalities and the sensitivity‐analysis mortality signal, should be regarded as hypothesis‐generating. Finally, although the overall cohort is large, the very small number of events for rare outcomes such as perforation yields wide confidence intervals and warrants cautious interpretation. Future prospective studies that incorporate validated frailty and life‐expectancy measures, cancer stage and cancer‐specific mortality are needed to refine the benefit–risk calculus of colonoscopy in the very elderly.

## 6 Conclusion

In this large propensity‐matched real‐world cohort study of adults aged 75–90 years, screening colonoscopy was associated with substantially higher detection of polyps and colorectal cancer across all follow‐up periods but did not reduce all‐cause mortality over 5 years. Yield remained higher after exclusion of pre‐index anemia and coded alarm features. Colonoscopy was associated with a modest late increase in perforation and higher emergency department utilization, but subsequent and therapeutic colonoscopy were not more frequent in the screened arm, so a simple surveillance cascade does not explain the late perforation signal. Lower hospitalization most likely reflects residual healthy‐user bias. These findings indicate that the principal value of colonoscopy in this age group lies in diagnostic yield rather than in a demonstrable survival benefit. The results support a personalized, shared decision‐making approach to screening colonoscopy in the very elderly, in which the potential for early detection is carefully weighed against competing mortality risks, procedural harms, and individual patient goals of care, rather than routine age‐based recommendations.

## Funding

The authors have nothing to report.

## Ethics Statement

This retrospective cohort study used de‐identified electronic health record and claims data from the TriNetX US Collaborative Network. Because all data are de‐identified and HIPAA‐compliant, the study was exempt from institutional review board approval. Informed consent was not required.

## Consent

The authors have nothing to report.

## Conflicts of Interest

The authors declare no conflicts of interest.

## Supporting information


**Table S1:** Query criteria for Cohort 1 (scope group 75–90).
**Table S2:** Query criteria for Cohort 2 (no scope group 75–90).
**Table S3:** Outcome definitions.
**Table S4a:** Propensity score matching—30‐day analysis.
**Table S4b:** Propensity score matching—1‐year analysis.
**Table S4c:** Propensity score matching—5‐year analysis.
**Tabe S5:** Clinical outcomes across sensitivity and subgroup analyses (5‐year follow‐up).
**Table S6:** Diagnostic yield after exclusion of pre‐index anemia and in the indication‐restricted screening cohort.
**Table S7:** Subsequent and therapeutic colonoscopy through 5 years.
**Figure S1:** Kaplan–Meier survival curves—strict definition sensitivity analysis (ages 75–85, CPT codes only).
**Figure S2:** Kaplan–Meier survival curves—female subgroup (ages 75–90).
**Figure S3:** Kaplan–Meier survival curves—male subgroup (ages 75–90).
**Figure S4:** Kaplan–Meier survival curves—age subgroup 75–79 Years.
**Figure S5:** Kaplan–Meier survival curves—age subgroup 80–84 Years.
**Figure S6:** Kaplan–Meier survival curves—age subgroup 85–90 Years.
**Figure S7:** Kaplan–Meier survival curves for emergency department visit, polyp detection, colorectal cancer and Mortality.
**Figure S8:** Forest plot for pooled effect of all clinical outcomes at 30‐day follow‐up.
**Figure S9:** Forest plot for pooled effect of all clinical outcomes at 1 year follow‐up.

## Data Availability

The data that support the findings of this study are available from TriNetX, but restrictions apply to the availability of these data, which were used under license for the current study and are not publicly available. Data are available from the corresponding author upon reasonable request and with permission of TriNetX.
