# Supplementary material for: Outcomes of Screening Colonoscopy in Older Adults: A Large Propensity‐Matched Real‐World Cohort Study
Source: JGH Open. 2026 Oct 4;10(10):e70479. doi: 10.1002/jgh3.70479 (PMC13635485; doi:10.1002/jgh3.70479)
Supplement: Supplementary file 1 [file JGH3-10-e70479-s001.docx]

**Supplementary Appendix**

**Supplemental Table S1:** Query Criteria for Cohort 1 (Scope Group 75–90)

**Supplemental Table S2:** Query Criteria for Cohort 2 (No Scope Group 75–90)

**Supplemental Table S3:** Outcome Definitions

**Supplemental Table S4a:** Propensity Score Matching – 30-Day Analysis

**Supplemental Table S4b:** Propensity Score Matching – 1-Year Analysis

**Supplemental Table S4c:** Propensity Score Matching – 5-Year Analysis **Supplemental Tabe S5: C**linical Outcomes Across Sensitivity and Subgroup Analyses (5-Year Follow-Up)

**Supplementary Table S6.** Diagnostic yield after exclusion of pre-index anemia and in the indication-restricted screening cohort

**Supplementary Table S7.** Subsequent and therapeutic colonoscopy through 5 years

**Supplementary Figure S1.** Kaplan–Meier Survival Curves — Strict Definition Sensitivity Analysis (Ages 75–85, CPT Codes Only)

**Supplementary Figure S2.** Kaplan–Meier Survival Curves — Female Subgroup (Ages 75–90)

**Supplementary Figure S3.** Kaplan–Meier Survival Curves — Male Subgroup (Ages 75–90)

**Supplementary Figure S4.** Kaplan–Meier Survival Curves — Age Subgroup 75–79 Years

**Supplementary Figure S5.** Kaplan–Meier Survival Curves — Age Subgroup 80–84 Years

**Supplementary Figure S6.** Kaplan–Meier Survival Curves — Age Subgroup 85–90 Years

**Supplementary Figure S7.** Kaplan–Meier Survival Curves for Emergency Department visit, Polyp Detection, Colorectal cancer and Mortality

**Supplementary Figure S8:** Forest plot for pooled effect of all clinical outcomes at 30-day follow-up **Supplementary Figure S9:** Forest plot for pooled effect of all clinical outcomes at 1 year follow-up

**Supplemental Table S1: Query Criteria for Cohort 1 (Scope Group 75–90)**

| **Ungrouped terms** | | | | | |
| --- | --- | --- | --- | --- | --- |
|  | must have |  | demographics | Age | Age (between 75 and 90 years (most recent occurrence)) |
|  |  |  |  |  |  |
| **Group 1** | | | | | |
|  | Group 1A Screening |  |  |  |  |
|  | must have | any of | diagnosis | UMLS:ICD10CM:Z12.12 | Encounter for screening for malignant neoplasm of rectum |
|  |  |  | diagnosis | UMLS:ICD10CM:Z12.11 | Encounter for screening for malignant neoplasm of colon |
|  |  |  |  | date constraint | The terms in this group occurred between Jan 1, 2013 and May 1, 2026 |
|  | Group 1B Scope |  |  |  |  |
|  | event relationship |  |  | Any instance of Scope occurred within 3 months on or before any instance of Screening |  |
|  | must have | any of | procedure | UMLS:HCPCS:G0121 | Colorectal cancer screening; colonoscopy on individual not meeting criteria for high risk (between 75 and 90 years old at event) |
|  |  |  | procedure | UMLS:CPT:45378 | Colonoscopy, flexible; diagnostic, including collection of specimen(s) by brushing or washing, when performed (between 75 and 90 years old at event) |
|  |  |  | procedure | UMLS:CPT:45380 | Colonoscopy, flexible; with biopsy, single or multiple (between 75 and 90 years old at event) |
|  |  |  | procedure | UMLS:CPT:45385 | Colonoscopy, flexible; with removal of tumor(s), polyp(s), or other lesion(s) by snare technique (between 75 and 90 years old at event) |
|  |  |  | procedure | UMLS:HCPCS:G0105 | Colorectal cancer screening; colonoscopy on individual at high risk (between 75 and 90 years old at event) |
|  |  |  |  |  |  |
| **Group 2** | | | | | |
|  | Group 2A Screening |  |  |  |  |
|  | must have | any of | diagnosis | UMLS:ICD10CM:Z12.12 | Encounter for screening for malignant neoplasm of rectum (between 75 and 90 years old at event) |
|  |  |  | diagnosis | UMLS:ICD10CM:Z12.11 | Encounter for screening for malignant neoplasm of colon (between 75 and 90 years old at event) |
|  |  |  |  | date constraint | The terms in this group occurred between Jan 1, 2013 and May 1, 2026 |
|  | Group 2B GI bleed |  |  |  |  |
|  | event relationship |  |  | Any instance of GI bleed occurred within 1 month and 1 day before any instance of Screening |  |
|  | cannot have |  | diagnosis | UMLS:ICD10CM:K92.0 | Hematemesis |
|  |  | or | diagnosis | UMLS:ICD10CM:K92.1 | Melena |
|  |  | or | diagnosis | UMLS:ICD10CM:K92.2 | Gastrointestinal hemorrhage, unspecified |
|  |  |  |  |  |  |
| **Group 3** | | | | | |
|  | Group 3A Colonoscopy |  |  |  |  |
|  | must have | any of | diagnosis | UMLS:ICD10CM:Z12.12 | Encounter for screening for malignant neoplasm of rectum (between 75 and 90 years old at event) |
|  |  |  | diagnosis | UMLS:ICD10CM:Z12.11 | Encounter for screening for malignant neoplasm of colon (between 75 and 90 years old at event) |
|  |  |  |  | date constraint | The terms in this group occurred between Jan 1, 2013 and May 1, 2026 |
|  | Group 3B polyp |  |  |  |  |
|  | event relationship |  |  | Any instance of polyp occurred at least 1 month before any instance of Colonoscopy |  |
|  | cannot have |  | diagnosis | UMLS:ICD10CM:K63.5 | Polyp of colon |
|  |  | or | diagnosis | UMLS:ICD10CM:Z86.010 | Personal history of colon polyps |
|  |  |  |  |  |  |
| **Group 4** | | | | | |
|  | Group 4A Scope |  |  |  |  |
|  | must have | any of | diagnosis | UMLS:ICD10CM:Z12.12 | Encounter for screening for malignant neoplasm of rectum (between 75 and 90 years old at event) |
|  |  |  | diagnosis | UMLS:ICD10CM:Z12.11 | Encounter for screening for malignant neoplasm of colon (between 75 and 90 years old at event) |
|  |  |  |  | date constraint | The terms in this group occurred between Jan 1, 2013 and May 1, 2026 |
|  | Group 4B Ca |  |  |  |  |
|  | event relationship |  |  | Any instance of Ca occurred at least 1 month before any instance of Scope |  |
|  | cannot have |  | diagnosis | UMLS:ICD10CM:C18 | Malignant neoplasm of colon |
|  |  | or | diagnosis | UMLS:ICD10CM:C19 | Malignant neoplasm of rectosigmoid junction |
|  |  | or | diagnosis | UMLS:ICD10CM:C20 | Malignant neoplasm of rectum |
|  |  |  |  |  |  |
| **Group 5** | | | | | |
|  | Group 5A Screening |  |  |  |  |
|  | must have | any of | diagnosis | UMLS:ICD10CM:Z12.12 | Encounter for screening for malignant neoplasm of rectum |
|  |  |  | diagnosis | UMLS:ICD10CM:Z12.11 | Encounter for screening for malignant neoplasm of colon |
|  |  |  |  | date constraint | The terms in this group occurred between Jan 1, 2013 and May 1, 2026 |
|  | Group 5B IBD |  |  |  |  |
|  | event relationship |  |  | Any instance of IBD occurred at least 1 month before any instance of Screening |  |
|  | cannot have |  | diagnosis | UMLS:ICD10CM:K51 | Ulcerative colitis |
|  |  | or | diagnosis | UMLS:ICD10CM:K50 | Crohn's disease [regional enteritis] |

Footnote: The anemia-excluded sensitivity cohort additionally required the absence of ICD-10-CM D64.9 before the screening encounter. The indication-restricted cohort additionally required the absence of D64.9 before screening and the absence of R19.5, R63.4, R10.9, and R19.4 in the 6 months before screening.

**Supplemental Table S2: Query Criteria for Cohort 2 (No Scope Group 75–90)**

| **Ungrouped terms** | | | | | |
| --- | --- | --- | --- | --- | --- |
|  | must have |  | demographics | Age | Age (between 75 and 90 years (most recent occurrence)) |
|  |  |  |  |  |  |
| **Group 1** | | | | | |
|  | Group 1A Screening |  |  |  |  |
|  | must have | any of | diagnosis | UMLS:ICD10CM:Z12.12 | Encounter for screening for malignant neoplasm of rectum |
|  |  |  | diagnosis | UMLS:ICD10CM:Z12.11 | Encounter for screening for malignant neoplasm of colon |
|  |  |  |  | date constraint | The terms in this group occurred between Jan 1, 2013 and May 1, 2026 |
|  | Group 1B Scope |  |  |  |  |
|  | event relationship |  |  | Any instance of Scope occurred within 3 months on or before any instance of Screening |  |
|  | cannot have |  | procedure | UMLS:CPT:45380 | Colonoscopy, flexible; with biopsy, single or multiple (between 75 and 90 years old at event) |
|  |  | or all of | procedure | UMLS:HCPCS:G0105 | Colorectal cancer screening; colonoscopy on individual at high risk (between 75 and 90 years old at event) |
|  |  |  | procedure | UMLS:HCPCS:G0121 | Colorectal cancer screening; colonoscopy on individual not meeting criteria for high risk (between 75 and 90 years old at event) |
|  |  | or | procedure | UMLS:CPT:45385 | Colonoscopy, flexible; with removal of tumor(s), polyp(s), or other lesion(s) by snare technique (between 75 and 90 years old at event) |
|  |  | or | procedure | UMLS:CPT:45378 | Colonoscopy, flexible; diagnostic, including collection of specimen(s) by brushing or washing, when performed (between 75 and 90 years old at event) |
|  |  |  |  |  |  |
| **Groups 2–5 (GI Bleed, Polyp, Cancer, IBD Exclusions)** | | | | | |
|  |  |  |  | Same exclusion criteria as Cohort 1 Groups 2B–5B applied identically |  |
|  |  |  |  |  |  |
| **Group 2B GI bleed – cannot have** | | | | | |
|  | cannot have |  | diagnosis | UMLS:ICD10CM:K92.0 | Hematemesis |
|  |  | or | diagnosis | UMLS:ICD10CM:K92.1 | Melena |
|  |  | or | diagnosis | UMLS:ICD10CM:K92.2 | Gastrointestinal hemorrhage, unspecified |
|  |  |  |  |  |  |
| **Group 3B polyp – cannot have (at least 1 month before index)** | | | | | |
|  | cannot have |  | diagnosis | UMLS:ICD10CM:K63.5 | Polyp of colon |
|  |  | or | diagnosis | UMLS:ICD10CM:Z86.010 | Personal history of colon polyps |
|  |  |  |  |  |  |
| **Group 4B Ca – cannot have (at least 1 month before index)** | | | | | |
|  | cannot have |  | diagnosis | UMLS:ICD10CM:C18 | Malignant neoplasm of colon |
|  |  | or | diagnosis | UMLS:ICD10CM:C19 | Malignant neoplasm of rectosigmoid junction |
|  |  | or | diagnosis | UMLS:ICD10CM:C20 | Malignant neoplasm of rectum |
|  |  |  |  |  |  |
| **Group 5B IBD – cannot have (at least 1 month before index)** | | | | | |
|  | cannot have |  | diagnosis | UMLS:ICD10CM:K51 | Ulcerative colitis |
|  |  | or | diagnosis | UMLS:ICD10CM:K50 | Crohn's disease [regional enteritis] |

Footnote: The anemia-excluded sensitivity cohort additionally required the absence of ICD-10-CM D64.9 before the screening encounter. The indication-restricted cohort additionally required the absence of D64.9 before screening and the absence of R19.5, R63.4, R10.9, and R19.4 in the 6 months before screening.

**Supplemental Table S3: Outcome Definitions**

| **Intestinal perforation** | | | | | |
| --- | --- | --- | --- | --- | --- |
|  |  |  |  |  |  |
| **Outcome definition** | | | | | |
|  |  |  | Diagnosis | UMLS:ICD10CM:K63.1 | Perforation of intestine (nontraumatic) |
|  |  |  |  |  |  |
| **Settings for the performed analyses** | | | | | |
|  |  |  | Kaplan - Meier survival analysis |  | including patients with outcome prior to the time window |
|  |  |  | Risk analysis |  | including patients with outcome prior to the time window |
|  |  |  |  |  |  |
| **Gastrointestinal bleeding** | | | | | |
|  |  |  |  |  |  |
| **Outcome definition** | | | | | |
|  |  |  | Diagnosis | UMLS:ICD10CM:K92.0 | Hematemesis |
|  |  |  | Diagnosis | UMLS:ICD10CM:K92.1 | Melena |
|  |  |  | Diagnosis | UMLS:ICD10CM:K92.2 | Gastrointestinal hemorrhage, unspecified |
|  |  |  | Diagnosis | UMLS:ICD10CM:K62.5 | Hemorrhage of anus and rectum |
|  |  |  |  |  |  |
| **Settings for the performed analyses** | | | | | |
|  |  |  | Kaplan - Meier survival analysis |  | including patients with outcome prior to the time window |
|  |  |  | Risk analysis |  | including patients with outcome prior to the time window |
|  |  |  | Number of instances analysis |  | including patients with outcome prior to the time window; excluding patients with zero outcomes; counts are grouped by date |
|  |  |  |  |  |  |
| **Electrolyte abnormality** | | | | | |
|  |  |  |  |  |  |
| **Outcome definition** | | | | | |
|  |  |  | Diagnosis | UMLS:ICD10CM:E86.0 | Dehydration |
|  |  |  | Diagnosis | UMLS:ICD10CM:E87.1 | Hypo-osmolality and hyponatremia |
|  |  |  | Diagnosis | UMLS:ICD10CM:E87.6 | Hypokalemia |
|  |  |  |  |  |  |
| **Settings for the performed analyses** | | | | | |
|  |  |  | Kaplan - Meier survival analysis |  | including patients with outcome prior to the time window |
|  |  |  | Risk analysis |  | including patients with outcome prior to the time window |
|  |  |  |  |  |  |
| **Hospitalization** | | | | | |
|  |  |  |  |  |  |
| **Outcome definition** | | | | | |
|  |  |  | Visit | UMLS:HL7V3.0:VisitType:IMP | Visit: Inpatient Encounter |
|  |  |  | Procedure | UMLS:SNOMED:183452005 | Emergency hospital admission |
|  |  |  |  |  |  |
| **Settings for the performed analyses** | | | | | |
|  |  |  | Kaplan - Meier survival analysis |  | including patients with outcome prior to the time window |
|  |  |  | Risk analysis |  | including patients with outcome prior to the time window |
|  |  |  | Number of instances analysis |  | including patients with outcome prior to the time window; excluding patients with zero outcomes; counts are grouped by date |
|  |  |  |  |  |  |
| **Emergency department visit** | | | | | |
|  |  |  |  |  |  |
| **Outcome definition** | | | | | |
|  |  |  | Procedure | UMLS:CPT:1013711 | Emergency Department Services |
|  |  |  | Visit | UMLS:HL7V3.0:VisitType:EMER | Visit: Emergency |
|  |  |  | Procedure | UMLS:CPT:99283 | Emergency department visit for the evaluation and management of a patient |
|  |  |  |  |  |  |
| **Settings for the performed analyses** | | | | | |
|  |  |  | Kaplan - Meier survival analysis |  | including patients with outcome prior to the time window |
|  |  |  | Risk analysis |  | including patients with outcome prior to the time window |
|  |  |  | Number of instances analysis |  | including patients with outcome prior to the time window; excluding patients with zero outcomes; counts are grouped by date |
|  |  |  |  |  |  |
| **Polyp detection** | | | | | |
|  |  |  |  |  |  |
| **Outcome definition** | | | | | |
|  |  |  | Diagnosis | UMLS:ICD10CM:K63.5 | Polyp of colon |
|  |  |  | Diagnosis | UMLS:ICD10CM:D12.0 | Benign neoplasm of cecum |
|  |  |  | Diagnosis | UMLS:ICD10CM:D12.2 | Benign neoplasm of ascending colon |
|  |  |  | Diagnosis | UMLS:ICD10CM:D12.3 | Benign neoplasm of transverse colon |
|  |  |  | Diagnosis | UMLS:ICD10CM:D12.4 | Benign neoplasm of descending colon |
|  |  |  | Diagnosis | UMLS:ICD10CM:D12.5 | Benign neoplasm of sigmoid colon |
|  |  |  | Diagnosis | UMLS:ICD10CM:D12.6 | Benign neoplasm of colon, unspecified |
|  |  |  | Diagnosis | UMLS:ICD10CM:D12.7 | Benign neoplasm of rectosigmoid junction |
|  |  |  | Diagnosis | UMLS:ICD10CM:D12.8 | Benign neoplasm of rectum |
|  |  |  |  |  |  |
| **Settings for the performed analyses** | | | | | |
|  |  |  | Risk analysis |  | including patients with outcome prior to the time window |
|  |  |  | Kaplan - Meier survival analysis |  | including patients with outcome prior to the time window |
|  |  |  |  |  |  |
| **Colorectal cancer** | | | | | |
|  |  |  |  |  |  |
| **Outcome definition** | | | | | |
|  |  |  | Diagnosis | UMLS:ICD10CM:C18 | Malignant neoplasm of colon |
|  |  |  | Diagnosis | UMLS:ICD10CM:C19 | Malignant neoplasm of rectosigmoid junction |
|  |  |  | Diagnosis | UMLS:ICD10CM:C20 | Malignant neoplasm of rectum |
|  |  |  |  |  |  |
| **Settings for the performed analyses** | | | | | |
|  |  |  | Kaplan - Meier survival analysis |  | including patients with outcome prior to the time window |
|  |  |  | Risk analysis |  | including patients with outcome prior to the time window |
|  |  |  |  |  |  |
| **Mortality** | | | | | |
|  |  |  |  |  |  |
| **Outcome definition** | | | | | |
|  |  |  | Demographics | Deceased | Deceased |
|  |  |  |  |  |  |
| **Settings for the performed analyses** | | | | | |
|  |  |  | Risk analysis |  | including patients with outcome prior to the time window |
|  |  |  | Kaplan - Meier survival analysis |  | including patients with outcome prior to the time window |
| Any subsequent colonoscopy | | | | | |
|  |  |  |  | CPT 45378, 45380, 45385; HCPCS G0105, G0121 |  |
| Therapeutic colonoscopy | | | | | |
|  |  |  |  | CPT 45380 (biopsy); CPT 45385 (snare polypectomy) |  |

**Supplemental Table S4a: Propensity Score Matching – 30-Day Analysis**

Cohort 1 and cohort 2 patient count before and after propensity score matching

| **Cohort** | | **Patient count before matching** | **Patient count after matching** |
| --- | --- | --- | --- |
| 1 - Scope group 75-90 | | 58,322 | 58,315 |
| 2 - No scope 75-90 | | 248,087 | 58,315 |
| Propensity score density function – Before and after matching (cohort 1 – purple, cohort 2 – green) | | | |
|  | Before matching | | After matching |
|  |  | |  |

**Supplemental Table S4b: Propensity Score Matching – 1-Year Analysis**

Cohort 1 and cohort 2 patient count before and after propensity score matching

| **Cohort** | | **Patient count before matching** | **Patient count after matching** |
| --- | --- | --- | --- |
| 1 - Scope group 75-90 | | 61,498 | 61,497 |
| 2 - No scope 75-90 | | 252,720 | 61,497 |
| Propensity score density function – Before and after matching (cohort 1 – purple, cohort 2 – green) | | | |
|  | Before matching | | After matching |
|  |  | |  |

**Supplemental Table S4c: Propensity Score Matching – 5-Year Analysis**

Cohort 1 and cohort 2 patient count before and after propensity score matching

| **Cohort** | | **Patient count before matching** | **Patient count after matching** |
| --- | --- | --- | --- |
| 1 - Scope group 75-90 | | 69,822 | 69,821 |
| 2 - No scope 75-90 | | 272,500 | 69,821 |
| Propensity score density function – Before and after matching (cohort 1 – purple, cohort 2 – green) | | | |
|  | Before matching | | After matching |
|  |  | |  |

**Supplementary Table S5: Clinical Outcomes Across Sensitivity and Subgroup Analyses - 5-Year Follow-Up**

| **Strict Definition Sensitivity Analysis (Ages 75–85, CPT Codes Only) (n = 37,434 per group after 1:1 PSM; 5-year follow-up)** | | | | |
| --- | --- | --- | --- | --- |
| Note: Colonoscopy defined by CPT codes only (45378/45380/45385); HCPCS G0105/G0121 excluded; age restricted to 75–85 years | | | | |
| **Outcome** | **Scope Group (n = 37,434)** | **No Scope Group (n = 37,434)** | **HR (95% CI)** | **p-value** |
| Intestinal Perforation | 70 (0.19%) | 67 (0.18%) | 1.066 (0.762, 1.490) | 0.710 |
| Gastrointestinal Bleeding | 2,024 (5.41%) | 1,990 (5.32%) | 1.039 (0.977, 1.105) | 0.226 |
| Electrolyte Abnormality | 5,461 (14.59%) | 5,214 (13.93%) | 1.076 (1.036, 1.117) | <0.001 |
| Hospitalization | 9,846 (26.30%) | 9,764 (26.09%) | 1.022 (0.994, 1.051) | 0.121 |
| Emergency Department Visit | 14,343 (38.32%) | 12,442 (33.24%) | 1.255 (1.225, 1.286) | <0.001 |
| Polyp Detection | 6,191 (16.54%) | 2,875 (7.68%) | 2.349 (2.247, 2.455) | <0.001 |
| Colorectal Cancer | 528 (1.41%) | 272 (0.73%) | 1.996 (1.725, 2.311) | <0.001 |
| All-Cause Mortality | 2,123 (5.67%) | 2,015 (5.38%) | 1.075 (1.012, 1.143) | 0.019 |
|  | | | | |
| **Female Subgroup (Ages 75–90) (n = 37,558 per group after 1:1 PSM; 5-year follow-up)** | | | | |
| **Outcome** | **Scope Group (n = 37,558)** | **No Scope Group (n = 37,558)** | **HR (95% CI)** | **p-value** |
| Intestinal Perforation | 94 (0.25%) | 87 (0.23%) | 1.094 (0.817, 1.464) | 0.548 |
| Gastrointestinal Bleeding | 2,385 (6.35%) | 2,325 (6.19%) | 1.037 (0.979, 1.098) | 0.216 |
| Electrolyte Abnormality | 6,555 (17.45%) | 6,624 (17.64%) | 1.001 (0.968, 1.036) | 0.942 |
| Hospitalization | 10,188 (27.13%) | 10,676 (28.43%) | 0.944 (0.919, 0.970) | <0.001 |
| Emergency Department Visit | 15,554 (41.41%) | 13,935 (37.11%) | 1.194 (1.167, 1.222) | <0.001 |
| Polyp Detection | 4,882 (13.00%) | 2,536 (6.75%) | 2.034 (1.938, 2.134) | <0.001 |
| Colorectal Cancer | 447 (1.19%) | 289 (0.77%) | 1.574 (1.357, 1.825) | <0.001 |
| All-Cause Mortality | 1,872 (4.98%) | 1,950 (5.19%) | 0.969 (0.909, 1.032) | 0.327 |
|  | | | | |
| **Male Subgroup (Ages 75–90) (n = 33,270 per group after 1:1 PSM; 5-year follow-up)** | | | | |
| **Outcome** | **Scope Group (n = 33,270)** | **No Scope Group (n = 33,270)** | **HR (95% CI)** | **p-value** |
| Intestinal Perforation | 65 (0.20%) | 68 (0.20%) | 0.974 (0.693, 1.368) | 0.878 |
| Gastrointestinal Bleeding | 2,104 (6.32%) | 2,116 (6.36%) | 1.011 (0.952, 1.074) | 0.714 |
| Electrolyte Abnormality | 4,805 (14.44%) | 4,717 (14.18%) | 1.038 (0.997, 1.081) | 0.066 |
| Hospitalization | 9,639 (28.97%) | 9,827 (29.54%) | 0.980 (0.953, 1.008) | 0.161 |
| Emergency Department Visit | 13,584 (40.83%) | 12,144 (36.50%) | 1.205 (1.176, 1.235) | <0.001 |
| Polyp Detection | 5,064 (15.22%) | 2,955 (8.88%) | 1.827 (1.746, 1.912) | <0.001 |
| Colorectal Cancer | 448 (1.35%) | 278 (0.84%) | 1.652 (1.422, 1.919) | <0.001 |
| All-Cause Mortality | 2,634 (7.92%) | 2,635 (7.92%) | 1.019 (0.966, 1.076) | 0.491 |
|  | | | | |
| **Age Subgroup 75–79 Years (n = 24,452 per group after 1:1 PSM; 5-year follow-up)** | | | | |
| **Outcome** | **Scope Group (n = 24,452)** | **No Scope Group (n = 24,452)** | **HR (95% CI)** | **p-value** |
| Intestinal Perforation | 27 (0.11%) | 30 (0.12%) | 0.919 (0.547, 1.546) | 0.751 |
| Gastrointestinal Bleeding | 770 (3.15%) | 752 (3.07%) | 1.047 (0.947, 1.158) | 0.371 |
| Electrolyte Abnormality | 2,307 (9.43%) | 2,243 (9.17%) | 1.054 (0.994, 1.117) | 0.076 |
| Hospitalization | 4,290 (17.55%) | 4,430 (18.12%) | 0.985 (0.945, 1.028) | 0.489 |
| Emergency Department Visit | 6,732 (27.53%) | 5,898 (24.12%) | 1.212 (1.171, 1.255) | <0.001 |
| Polyp Detection | 3,535 (14.46%) | 1,509 (6.17%) | 2.560 (2.410, 2.719) | <0.001 |
| Colorectal Cancer | 208 (0.85%) | 123 (0.50%) | 1.739 (1.391, 2.173) | <0.001 |
| All-Cause Mortality | 503 (2.06%) | 553 (2.26%) | 0.928 (0.823, 1.048) | 0.228 |
|  | | | | |
| **Age Subgroup 80–84 Years (n = 29,147 per group after 1:1 PSM; 5-year follow-up)** | | | | |
| **Outcome** | **Scope Group (n = 29,147)** | **No Scope Group (n = 29,147)** | **HR (95% CI)** | **p-value** |
| Intestinal Perforation | 74 (0.25%) | 80 (0.27%) | 0.934 (0.681, 1.282) | 0.673 |
| Gastrointestinal Bleeding | 1,994 (6.84%) | 2,012 (6.90%) | 0.998 (0.938, 1.062) | 0.945 |
| Electrolyte Abnormality | 5,123 (17.58%) | 5,092 (17.47%) | 1.015 (0.976, 1.055) | 0.467 |
| Hospitalization | 8,905 (30.55%) | 8,922 (30.61%) | 0.988 (0.960, 1.018) | 0.434 |
| Emergency Department Visit | 13,411 (46.01%) | 11,835 (40.61%) | 1.210 (1.180, 1.240) | <0.001 |
| Polyp Detection | 4,024 (13.81%) | 2,507 (8.60%) | 1.683 (1.601, 1.769) | <0.001 |
| Colorectal Cancer | 351 (1.20%) | 215 (0.74%) | 1.661 (1.402, 1.968) | <0.001 |
| All-Cause Mortality | 2,010 (6.90%) | 2,007 (6.89%) | 1.010 (0.949, 1.074) | 0.754 |
|  | | | | |
| **Age Subgroup 85–90 Years † (n = 18,625 per group after 1:1 PSM; 5-year follow-up)** | | | | |
| **Outcome** | **Scope Group (n = 18,625)** | **No Scope Group (n = 18,625)** | **HR (95% CI)** | **p-value** |
| Intestinal Perforation | 60 (0.32%) | 60 (0.32%) | 1.000 (0.699, 1.430) | 1.000 |
| Gastrointestinal Bleeding | 1,788 (9.60%) | 1,788 (9.60%) | 1.000 (0.937, 1.068) | 1.000 |
| Electrolyte Abnormality | 4,100 (22.02%) | 4,100 (22.02%) | 1.000 (0.958, 1.044) | 1.000 |
| Hospitalization | 6,761 (36.30%) | 6,761 (36.30%) | 1.000 (0.967, 1.034) | 1.000 |
| Emergency Department Visit | 9,628 (51.69%) | 9,628 (51.69%) | 1.000 (0.972, 1.029) | 1.000 |
| Polyp Detection | 2,466 (13.24%) | 2,466 (13.24%) | 1.000 (0.946, 1.057) | 1.000 |
| Colorectal Cancer | 346 (1.86%) | 346 (1.86%) | 1.000 (0.862, 1.161) | 1.000 |
| All-Cause Mortality | 2,068 (11.10%) | 2,068 (11.10%) | 1.000 (0.941, 1.063) | 1.000 |
|  | | | | |

† In the 85–90 years subgroup, both cohorts had identical patient counts (n = 18,625) prior to propensity score matching, resulting in no between-group variation; CI, confidence interval; HR, hazard ratio; PSM, propensity score matching; CPT, Current Procedural Terminology; HCPCS, Healthcare Common Procedure Coding System.

**Supplementary Table S6.** Diagnostic yield after exclusion of pre-index anemia and in the indication-restricted screening cohort

Panel A. Anemia-excluded cohort (pre-index ICD-10-CM D64.9 excluded). US Collaborative Network, 73 healthcare organizations. Matched n = 70,399 per group.

| **Follow-up** | **Outcome** | **Colonoscopy, n (%)** | **No colonoscopy, n (%)** | **HR (95% CI)** | **Log-rank p** |
| --- | --- | --- | --- | --- | --- |
| 30 days | Polyp detection | 4,809 (6.83) | 1,446 (2.05) | 3.51 (3.31–3.72) | <0.001 |
| 30 days | Colorectal cancer | 406 (0.58) | 110 (0.16) | 3.81 (3.09–4.70) | <0.001 |
| 1 year | Polyp detection | 7,068 (10.04) | 4,300 (6.11) | 1.75 (1.69–1.82) | <0.001 |
| 1 year | Colorectal cancer | 609 (0.87) | 311 (0.44) | 2.02 (1.76–2.32) | <0.001 |
| 5 years | Polyp detection | 9,502 (13.50) | 5,870 (8.34) | 1.72 (1.66–1.78) | <0.001 |
| 5 years | Colorectal cancer | 797 (1.13) | 539 (0.77) | 1.52 (1.36–1.69) | <0.001 |

Panel B. Indication-restricted cohort. Same exclusions as the primary analysis plus pre-index anemia (D64.9) and, in the 6 months before screening, fecal abnormalities (R19.5), unexplained weight loss (R63.4), unspecified abdominal pain (R10.9), and change in bowel habit (R19.4). US Collaborative Network, 70 healthcare organizations. Matched n = 54,300 per group.

| **Follow-up** | **Outcome** | **Colonoscopy, n (%)** | **No colonoscopy, n (%)** | **HR (95% CI)** | **Log-rank p** |
| --- | --- | --- | --- | --- | --- |
| 30 days | Polyp detection | 3,008 (5.54) | 1,050 (1.93) | 3.02 (2.81–3.24) | <0.001 |
| 30 days | Colorectal cancer | 256 (0.47) | 97 (0.18) | 2.74 (2.17–3.47) | <0.001 |
| 1 year | Polyp detection | 4,660 (8.58) | 3,081 (5.67) | 1.61 (1.54–1.68) | <0.001 |
| 1 year | Colorectal cancer | 392 (0.72) | 230 (0.42) | 1.77 (1.50–2.08) | <0.001 |
| 5 years | Polyp detection | 6,515 (12.00) | 4,327 (7.97) | 1.59 (1.53–1.66) | <0.001 |
| 5 years | Colorectal cancer | 535 (0.99) | 417 (0.77) | 1.32 (1.16–1.50) | <0.001 |

Footnote: HR, hazard ratio; CI, confidence interval. Analyses used 1:1 propensity-score matching on the same covariate set as the primary analysis. These extracts are not nested in Table 2 (primary analysis: 65 healthcare organizations). Percentages use the matched denominator of each panel.

**Supplementary Table S7.** Subsequent and therapeutic colonoscopy through 5 years.

| **Window** | **Outcome** | **Colonoscopy, n (%)** | **No colonoscopy, n (%)** | **HR (95% CI)** | **Log-rank p** |
| --- | --- | --- | --- | --- | --- |
| Day 1–1825 | Any colonoscopy | 5,137 (6.63) | 6,214 (8.02) | 0.83 (0.80–0.86) | <0.001 |
| Day 31–1825 | Any colonoscopy | 4,472 (5.77) | 4,824 (6.22) | 0.93 (0.90–0.97) | 0.001 |
| Day 31–1825 | Therapeutic colonoscopy (CPT 45380 or 45385) | 3,303 (4.26) | 3,389 (4.37) | 0.99 (0.94–1.03) | 0.567 |

**Footnote:** The day 31–1825 window is the primary subsequent-procedure analysis because day 1–30 may capture coding of the index examination. Any colonoscopy used the same CPT/HCPCS set as the exposure definition (45378, 45380, 45385, G0105, G0121). Tests of the proportional-hazards assumption were violated for these outcomes (platform χ² p<0.001); HRs should be read as average effects over follow-up. This extract is not nested in Table 2.

**Supplementary Figures. Kaplan–Meier Survival Curves for Sensitivity and Subgroup Analyses**

| **Supplementary Figure S1.** Kaplan–Meier Survival Curves — Strict Definition Sensitivity Analysis (Ages 75–85, CPT Codes Only) | |
| --- | --- |
| **A. Intestinal Perforation**   | **B. Gastrointestinal Bleeding**   |
| **C. Electrolyte Abnormality**   | **D. Hospitalization**   |
| **E. Emergency Department Visit**   | **F. Polyp Detection**   |
| **G. Colorectal Cancer**   | **H. All-Cause Mortality**   |

| **Supplementary Figure S2.** Kaplan–Meier Survival Curves — Female Subgroup (Ages 75–90) | |
| --- | --- |
| **A. Intestinal Perforation**   | **B. Gastrointestinal Bleeding**   |
| **C. Electrolyte Abnormality**   | **D. Hospitalization**   |
| **E. Emergency Department Visit**   | **F. Polyp Detection**   |
| **G. Colorectal Cancer**   | **H. All-Cause Mortality**   |

| **Supplementary Figure S3.** Kaplan–Meier Survival Curves — Male Subgroup (Ages 75–90) | |
| --- | --- |
| **A. Intestinal Perforation**   | **B. Gastrointestinal Bleeding**   |
| **C. Electrolyte Abnormality**   | **D. Hospitalization**   |
| **E. Emergency Department Visit**   | **F. Polyp Detection**   |
| **G. Colorectal Cancer**   | **H. All-Cause Mortality**   |

| **Supplementary Figure S4.** Kaplan–Meier Survival Curves — Age Subgroup 75–79 Years | |
| --- | --- |
| **A. Intestinal Perforation**   | **B. Gastrointestinal Bleeding**   |
| **C. Electrolyte Abnormality**   | **D. Hospitalization**   |
| **E. Emergency Department Visit**   | **F. Polyp Detection**   |
| **G. Colorectal Cancer**   | **H. All-Cause Mortality**   |

| **Supplementary Figure S5.** Kaplan–Meier Survival Curves — Age Subgroup 80–84 Years | |
| --- | --- |
| **A. Intestinal Perforation**   | **B. Gastrointestinal Bleeding**   |
| **C. Electrolyte Abnormality**   | **D. Hospitalization**   |
| **E. Emergency Department Visit**   | **F. Polyp Detection**   |
| **G. Colorectal Cancer**   | **H. All-Cause Mortality**   |

| **Supplementary Figure S6.** Kaplan–Meier Survival Curves — Age Subgroup 85–90 Years | |
| --- | --- |
| **A. Intestinal Perforation**   | **B. Gastrointestinal Bleeding**   |
| **C. Electrolyte Abnormality**   | **D. Hospitalization**   |
| **E. Emergency Department Visit**   | **F. Polyp Detection**   |
| **G. Colorectal Cancer**   | **H. All-Cause Mortality**   |

† In the 85–90 years subgroup, both cohorts contained identical patient counts (n = 18,625) prior to propensity score matching, resulting in no between-group variation; curves are therefore identical and results should be interpreted with caution.

**Supplementary Figure S7.** Kaplan–Meier Survival Curves for Emergency Department visit, Polyp Detection, Colorectal cancer and Mortality

**Supplementary Figure S8: Forest plot for pooled effect of all clinical outcomes at 30-day follow-up**

**Supplementary Figure S9: Forest plot for pooled effect of all clinical outcomes at 1 year follow-up**
